# Chemically Synthesized Iron-Oxide-Based Pure Negative Electrode for Solid-State Asymmetric Supercapacitor Devices

**DOI:** 10.3390/ma15176133

**Published:** 2022-09-03

**Authors:** A. A. Yadav, Y. M. Hunge, Seongjun Ko, Seok-Won Kang

**Affiliations:** 1 Department of Automotive Engineering, Yeungnam University, 280 Daehak-ro, Gyeongsan 38541, Korea; 2 Division of Biotechnology, Daegu Gyeongbuk Institute of Science and Technology (DGIST), Daegu 42988, Korea

**Keywords:** chemical bath deposition, α-Fe_2_O_3_, Co_3_O_4_, supercapacitor device

## Abstract

Among energy storage devices, supercapacitors have received considerable attention in recent years owing to their high-power density and extended cycle life. Researchers are currently making efforts to improve energy density using different asymmetric cell configurations, which may provide a wider potential window. Many studies have been conducted on positive electrodes for asymmetric supercapacitor devices; however, studies on negative electrodes have been limited. In this study, iron oxides with different morphologies were synthesized at various deposition temperatures using a simple chemical bath deposition method. A nanosphere-like morphology was obtained for α-Fe_2_O_3_. The obtained specific capacitance (*C_s_*) of α-Fe_2_O_3_ was 2021 F/g at a current density of 4 A/g. The negative electrode showed an excellent capacitance retention of 96% over 5000 CV cycles. The fabricated asymmetric solid-state supercapacitor device based on α-Fe_2_O_3_-NF//Co_3_O_4_-NF exhibited a *C_s_* of 155 F/g and an energy density of 21 Wh/kg at 4 A/g.

## 1. Introduction

Currently, energy storage and conversion are the major issues in sustainable development, and renewable energy sources are sufficient to fulfill the increasing demand for global energy [1]. In the case of conventional energy storage devices, few limitations exist in the development of energy storage devices with good performance and efficiency [2]. Compared to conventional energy storage devices, supercapacitors are emerging energy storage devices with good cyclic stability, a rapid charge–discharge rate with high-power density, enhanced temperature range, and long cycle life, which may meet the ever-growing demand for energy storage devices [3]. Depending on the charge storage mechanism, supercapacitors are mainly categorized into two types: (1) electric double-layer (EDLC) supercapacitors and (2) pseudocapacitors [4]. EDLCs predominantly exhibit capacitance developed from charge accumulation (non-Faradaic) that occurs at the electrode–electrolyte interface [5]. In pseudocapacitors, pseudocapacitance is mainly caused by the redox reactions that occur in electrolytes and the electrically active surface of the electrode material [6]. In particular, transition metal oxides and polymers have predominantly been studied as pseudocapacitor materials [7]. The transition metal oxides, such as NiO, Co_3_O_4_, and MnO_2_, and double metal oxides, such as MCo_2_O_4_ (M = Mn, Ni, Fe), are used as positive electrodes [7,8,9,10,11]. However, in the case of an asymmetric solid-state supercapacitor (ASC) device, the performance depends on both positive and negative electrodes. The capacitance of the cell was measured using the following formula [12]:

(1) $$\frac{1}{C}=\frac{1}{C_{+}}+\frac{1}{C_{-}}$$

where *C*_+_ and *C*_−_ are the specific capacitances (*C_s_*) of the anode and cathode, respectively. According to a study, the *C_s_* values observed in the case of the anode are higher than those of the cathode [13]. Therefore, identifying negative electrode materials with an enhanced electrochemical performance is challenging. Among the transition metal oxides, iron oxide has emerged as a potential negative electrode material. Fe_2_O_3_ is a naturally abundant metal oxide; it shows a more negative working potential with higher stability in alkaline electrolytes, in addition to a high theoretical capacity (3625 F/g) [9,14]. In the case of Fe_2_O_3_, the charges are mainly stored using Faradaic redox reactions that occur between the Fe^3+^ and Fe^2+^ ions and the electrolyte at the interface of the electrode and electrolyte [15,16,17,18]. Compared to other metallic oxides, α-Fe_2_O_3_ is a good pseudocapacitive material. This may be due to its special crystal structure, such as α-Fe_2_O_3_ (hematite), which has a steadier hexagonal-like crystal structure [19]. However, there are a few limitations in the usage of α-Fe_2_O_3_ as an electrode material, such as low electrical conductivity, and the particle size of α-Fe_2_O_3_ increases with the number of charge–discharge cycles [20].

Therefore, efforts are prevailing to design different nanostructured morphologies of iron oxide directly grown on conducting nickel foam (NF) substrates. The nanostructured porous morphology of α-Fe_2_O_3_ is suitable for improving the electrochemical performance because of the presence of a large number of active sites, which provide a large active surface area for charging–discharging reactions [18,19]. These active sites help in reducing the diffusion length and transfer resistance of electrons, which helps sustain a constant rate capability at high resistance [21]. Herein, α-Fe_2_O_3_ films were grown directly on NF substrates using a simple chemical bath deposition (CBD) method at different deposition temperatures. At 363 K, porous nonspherical particles of α-Fe_2_O_3_ were formed directly on the conducting NF substrate. For the fabrication of the ASC device, Co_3_O_4_-NF was used as the positive electrode, and the synthesized α-Fe_2_O_3_-NF electrode was used as the negative electrode. This ASC device showed a good energy density of 21.5 Wh k/g. The negative electrode show a good stability of 96% with improved Cs of 1037 F/g for KOH-based electrolytes.

## 2. Materials and Methods

### 2.1. Synthesis of Negative α-Fe_2_O_3_-NF Electrode

The α-Fe_2_O_3_ thin films were synthesized using a simple CBD method. Briefly, 0.1 M [Fe(NO_3_)_2_∙6H_2_O] (ferric nitrate) was dissolved in 25 mL of deionized water (DI) and kept under continuous stirring, and then 0.2 M [CO(NH_2_)_2_] (urea) was dissolved in the mixture and stirred for 25 min to form a uniform reaction mixture. A thoroughly cleaned NF substrate was then inserted into the solvent mixture. The beaker containing the reaction mixture was then kept in a constant-temperature water bath at different deposition temperatures, such as 353 (S1), 363 (S2), and 373 K (S3), for 3 h [22].

**Scheme 1:**
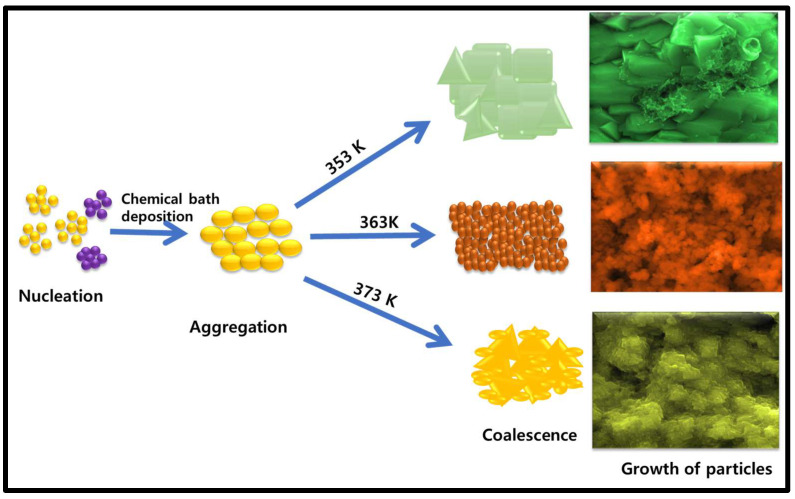
Schematic of synthesis of α-Fe_2_O_3_.

After deposition, the brown thin film was removed from the bath, washed repeatedly with DI water, and dried at room temperature for 5 h. A schematic of the synthesis of α-Fe_2_O_3_ is shown in Scheme 1. In addition, the brown Fe_2_O_3_ thin film confirmed the formation of α-Fe_2_O_3_ nanoparticles.

### 2.2. Synthesis of the Positive Co_3_O_4_-NF Electrode

In the facile synthesis of the positive electrode, Co_3_O_4_-NF was synthesized using a previously reported CBD method [23]. A reaction mixture of 0.1 M [Co(NO_3_)_2_] (cobalt nitrate) and 0.2 M [CO(NH_2_)_2_] (urea) was formed in 25 mL of DI water. This reaction mixture and the cleaned NF substrate were maintained at 363 K for 5 h. The as-deposited thin films were dried in air and annealed at 623 K.

## 3. Results and Discussion

### 3.1. Structural and Morphological Characterization of the Negative α-Fe_2_O_3_-NF Electrode

**Figure 1:**
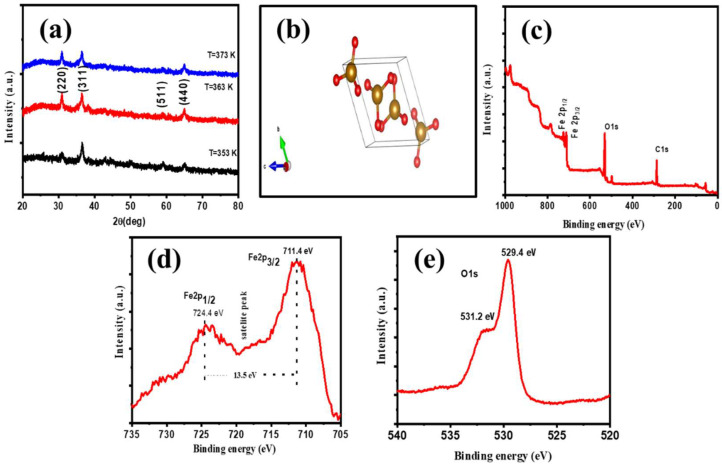
(**a**) XRD patterns of α-Fe_2_O_3_ at different deposition temperatures. (**b**) Crystal structure of α-Fe_2_O_3_. Brown and red balls represent Fe and O elements. XPS study for α-Fe_2_O_3_ (**c**) survey scan spectra, (**d**) Fe 2p core-level spectrum, and (**e**) O 1s core-level spectrum.

The XRD patterns of the S1, S2, and S3 thin films are shown in Figure 1a. The observed high-intensity peaks correspond to (012), (104), (110), (113), (024), (116), (214), and (300) planes and match well with the JCPDS card no. 79-0007. The α phase of the Fe_2_O_3_ thin film formation is confirmed by the peak position [24]. The different deposition temperatures affected the nucleation rate, surface structure, and growth direction of the α-Fe_2_O_3_ thin films. For the S2 sample, the observed high-intensity peaks correspond to the (013), (104), and (115) planes. Further, the intensities of the XRD peaks for the S1 sample decreased due to the incomplete growth of particles, while in the case of the S3 sample, the film peeled up from the NF substrate; therefore, it showed a low intensity [25]. The crystal structure of α-Fe_2_O_3_ was reproduced using the VISTA software, as shown in Figure 1b. The brown and red balls represent the Fe and O elements, respectively, and the CIF file was downloaded from the crystallography open database [26]. XPS analysis was performed to study the composition and bonding of α-Fe_2_O_3_. The survey scan spectra of α-Fe_2_O_3_ are shown in Figure 1c. The high-resolution survey scan spectra confirmed the presence of Fe 2p, O 1s, and C 1s. The Fe 2p spectrum shows two pronounced peaks positioned at 711.4 (Fe 2p_3/2_) and 724.4 eV (Fe 2p_1/2_), respectively (Figure 1d). The binding energy difference between the two peaks is 13.5 eV, which agrees well with the previously reported literature for the Fe 2p spectrum [27]. The two satellite peaks are present near the main peak at the binding energies of 719.2 and 732.4 eV, which shows that the oxidation phase is Fe^3+^. The O 1s core-level peak was resolved into two peaks at binding energies of 529.4 and 531.2 eV (Figure 1e) [28]. The peak at 529.4 eV was mainly due to lattice oxygen (FeO), and that at 531.2 eV was due to surface hydroxyl (–OH) [29,30].

**Figure 2:**
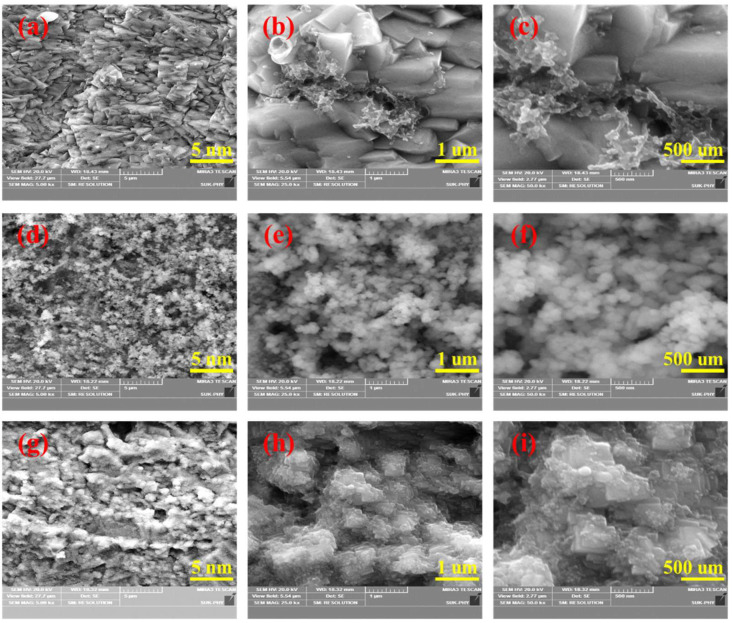
(**a**–**c**) SEM images of S1, (**d**–**f**) S2, and (**g**–**i**) S3 thin films at different magnifications.

**Figure 3:**
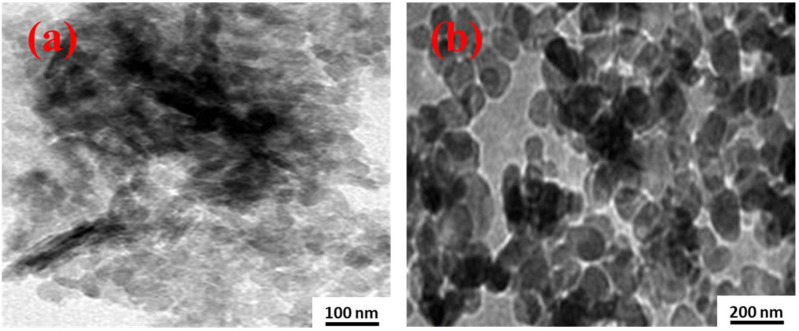
(**a**,**b**) TEM images of α-Fe_2_O_3_.

Figure 2 shows the surface morphologies of the α-Fe_2_O_3_ thin films (S1, S2, and S3). At low temperatures, the rhombohedra with connected edge-like morphologies were obtained for the S1 sample (Figure 2a–c). In addition, owing to the low reaction temperature, the rate of reaction was reduced, which affected the process of thin-film formation, such as nucleation, aggregation, and growth [31,32]. A further increase in deposition temperature resulted in nanosphere-like morphology, as shown in Figure 2d–f. The particle size was found in the range of 30–40 nm from the SEM observation. This nanosphere-like morphology mainly enhances the electrochemical properties. At higher temperatures, agglomeration of the particles occurred (Figure 2g–i). The morphology of α-Fe_2_O_3_ was further confirmed using TEM. Figure 3a,b show the TEM images of S2, which are in good agreement with the SEM data.

### 3.2. Electrochemical Performance of the α-Fe_2_O_3_-NF Electrode

**Figure 4:**
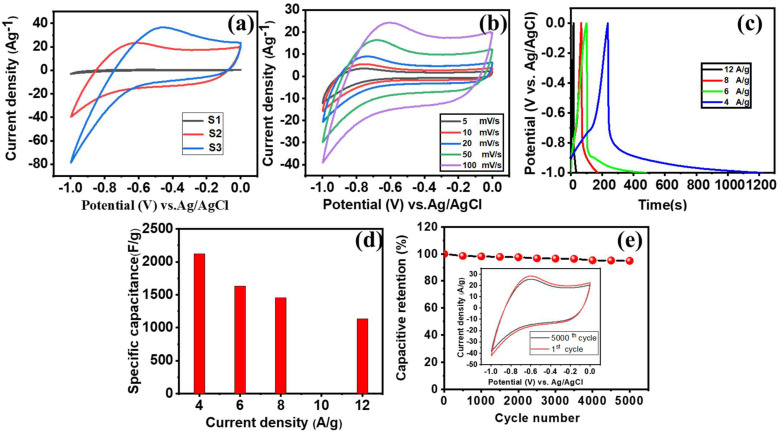
CV study for (**a**) S1, S2, and S3 samples, and (**b**) different scan rates; (**c**) GCD curves at different current densities; (**d**) plot of specific capacitance vs. current density; (**e**) plot of capacity retention vs. cycle number and inset shows the stability study at 5000 CV cycles for the α-Fe_2_O_3_ thin film.

**Figure A1:**
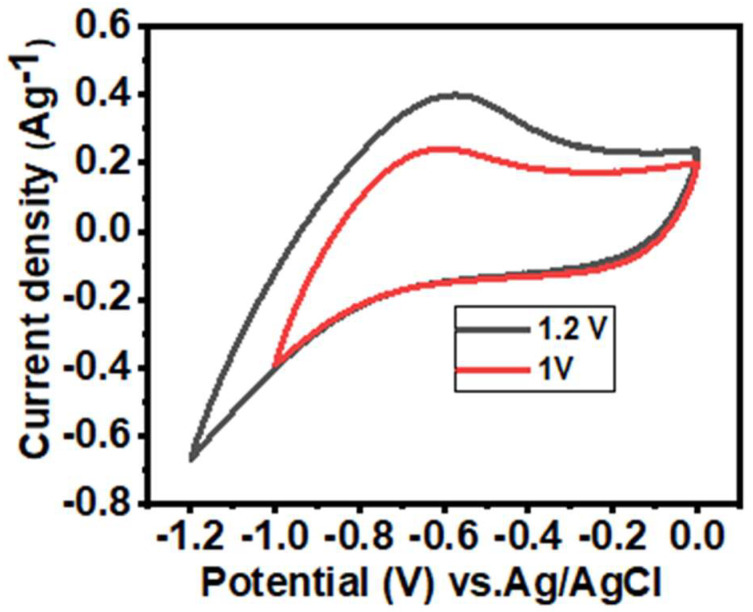
CV curves for α-Fe_2_O_3_-NF in −1 V and −1.2 V at 100 mV/s.

Figure 4a shows the CV curves of the S1, S2, and S3 samples at 100 mV/s. The area under the curve for the S2 sample was greater than that of the S1 and S3 samples. The electrochemical study for the negative α-Fe_2_O_3_-NF electrode(S2) was tested in a 2 M KOH electrolyte in the operating potential window range from 0.0 to −1.0 V Ag/AgCl. The CVs of the α-Fe_2_O_3_-NF electrodes were studied at various scan rates (Figure 4b). The CV curves for α-Fe_2_O_3_-NF at −1 and −1.2 V are shown in Figure A1. The shape of the CV curve was semi-rectangular, owing to the reversible redox reactions occurring between the Fe^3+^ and Fe^2+^ ions in the KOH electrolyte. This study substantiates the pseudocapacitive nature of the α-Fe_2_O_3_ electrode [32]. At lower scan rates, such as 5 mV/s, the available duration for the OH− ions in the electrolyte (KOH) to intercalate with the electrode material (α-Fe_2_O_3_-NF) is the maximum, which may be responsible for the enhancement of *C_s_*. The charging mechanism in the α-Fe_2_O_3_-NF electrode is shown in the following reaction [33,34]:

(2) $$Fe_{2}^{III}O_{3}+3H_{2}O+2e^{-}↔Fe^{II}{\left(OH\right)}_{2}+2OH^{-}$$

**Figure A2:**
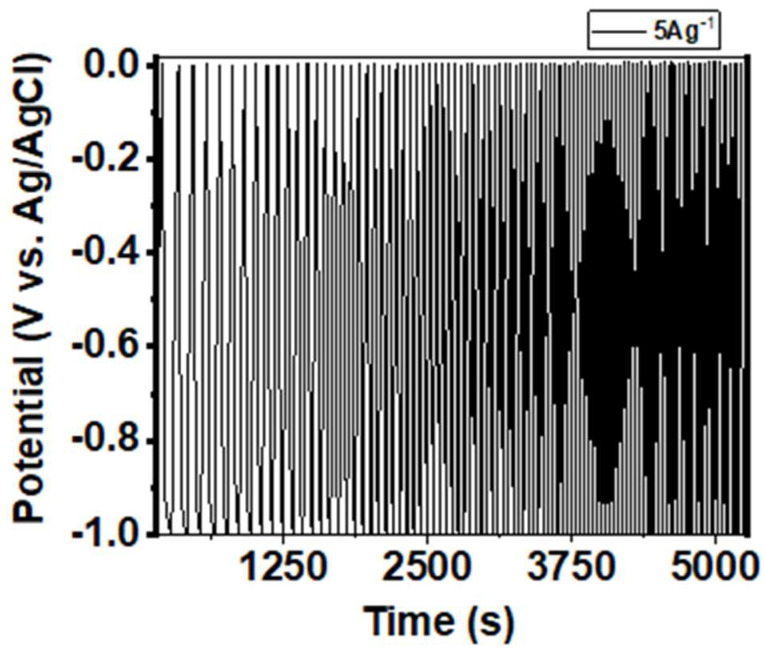
Stability test results for 5000 charge–discharge cycles at a current density of 5 A/g.

**Figure A3:**
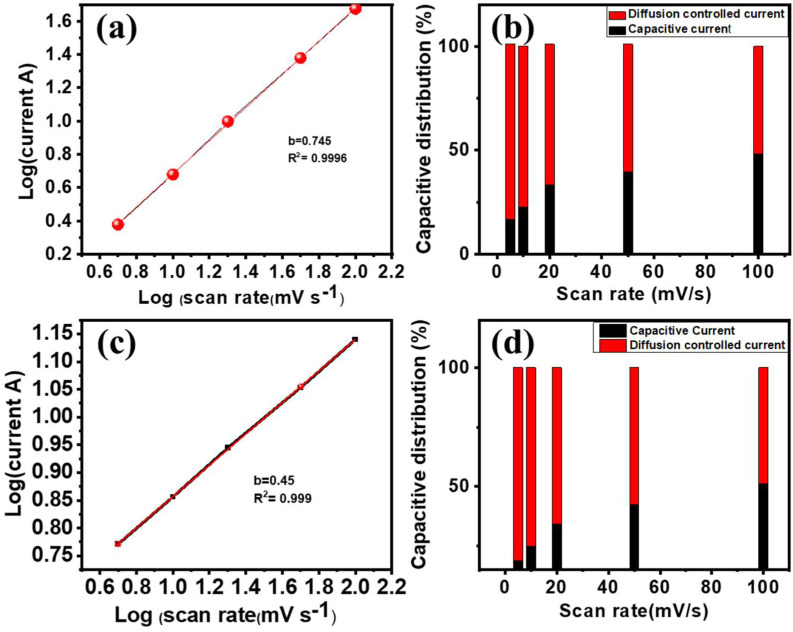
(**a**,**c**) Plot of current vs. scan rate in log-scale to calculate the b-value, (**b**,**d**) the plot of capacitive and diffusion-controlled current contributions with scan rates corresponding to Fe_2_O_3_ and Co_3_O_4_, respectively.

The GCD curves for α-Fe_2_O_3_ at various current densities in the operating window, ranged from 0.0 to −1.0 V Ag/AgCl, are shown in Figure 4c. The GCD curve shows the nonlinear nature of the discharge curve owing to the redox reactions occurring at the interface. The *C_s_* value of the α-Fe_2_O_3_ electrode at a current density of 4 A/g was 2125 F/g, which was calculated using Equation (A1) in the Appendix. The cyclic stability performance also decreased after 5000 GCD cycles, as shown in Figure A2. Figure 4d shows the variations in *C_s_* with various current densities. The inset of Figure 4e shows the stability of the α-Fe_2_O_3_-NF electrode, tested using CV cycling at 100 mV/s (scan rate) for 5000 CV cycles. Figure 4e shows the plot of capacity retention vs. cycle number, and the observed electrochemical stability of the α-Fe_2_O_3_-NF electrode was 96%. The improved performance of the α-Fe_2_O_3_-NF electrode was predominantly due to its porous morphology, which decreases the diffusion length and increases the rate capability. In addition, the α-Fe_2_O_3_ nanospheres were strongly attached to the conductive NF substrate, which prevented the loss of the active material during the cyclic stability performance study. The electrochemical performance of the α-Fe_2_O_3_-NF electrode is given in Table A1 (Appendix A). Therefore, the nanosphere-like α-Fe_2_O_3_-NF electrode is suitable for use as a negative electrode in the fabrication of ASC devices. The calculated b-value is 0.7 at the cathodic potential, which is close to 0.5, rather than 1, proving that the charge storage mechanism originates from the dominant diffusion-controlled process, as shown in Figure A3a. In addition, Figure A3b shows the capacitive and diffusive current-controlled distribution for the α-Fe_2_O_3_ electrode [35].

### 3.3. Structural and Morphological Characterization of the Positive Co_3_O_4_-NF Electrode

**Figure 5:**
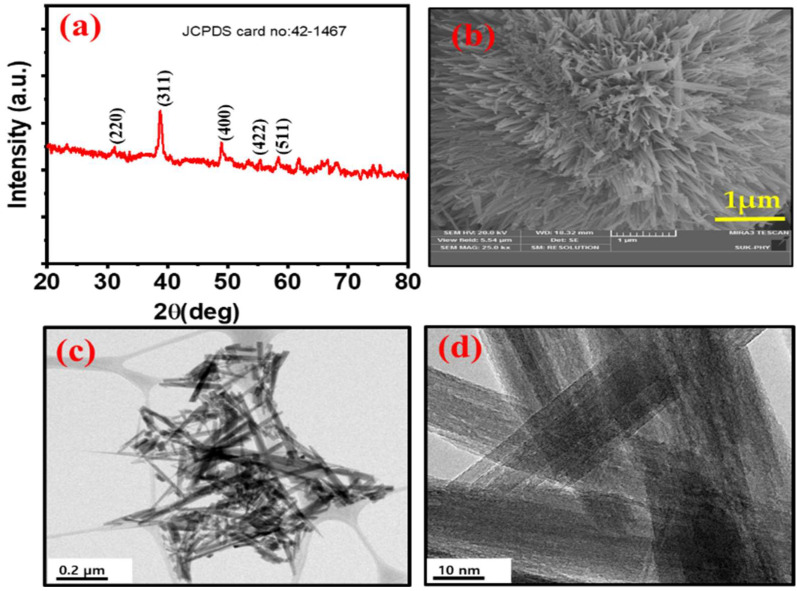
(**a**) XRD pattern, (**b**) SEM images, and (**c**,**d**) TEM images for the Co_3_O_4_ thin film.

A nanowire-like Co_3_O_4_-NF thin film was synthesized using a facile CBD method. The XRD pattern of the deposited Co_3_O_4_ is shown in Figure 5a. The peaks observed at 31.4°, 36.9°, 44.9°, 59.4°, and 65.3° are indexed to planes corresponding to (220), (311), (400), (422), and (511), respectively, according to JCPDS card no:42-1467. Figure 5b shows the FE-SEM image for Co_3_O_4_-NF. The observed nanowire-like morphology is uniformly distributed on the surface of the NF substrate. The nanowire-like morphology of Co_3_O_4_-NF provides less resistance value and porous structure, which improves the reaction rate [36]. Figure 5c,d show the TEM images of the Co_3_O_4_-NF electrode, which substantiates its nanowire-like nature.

### 3.4. Electrochemical Performance of the Positive Co_3_O_4_-NF Electrode

**Figure 6:**
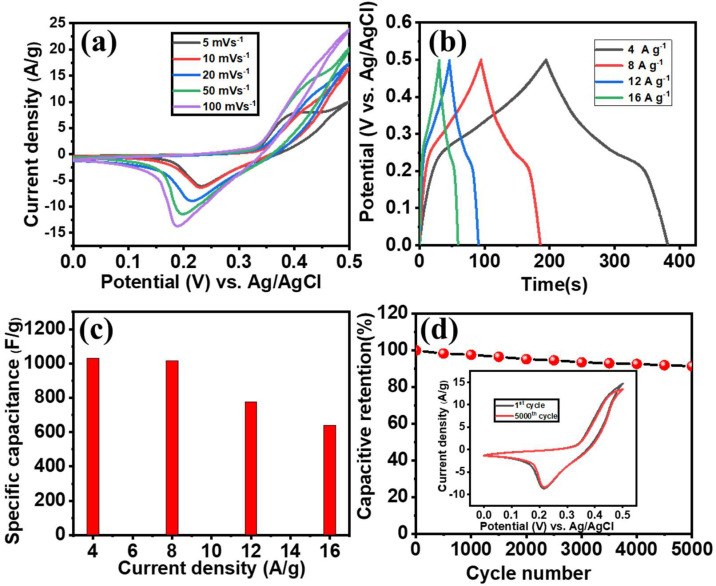
(**a**) CV curves at different scan rates, (**b**) GCD curves at different current densities, (**c**) plot of specific capacitance vs. current density, (**d**) plot of capacity retention vs. cycle number, and the inset shows the stability study at 5000 CV cycles for the Co_3_O_4_-NF thin film.

The electrochemical study of the Co_3_O_4_-NF electrode was conducted in a 2 M KOH electrolyte. The CV curves of the Co_3_O_4_-NF electrode are shown in Figure 6a. The CV curves for the Co_3_O_4_-NF electrode show the presence of a pair of redox peaks, which originate from the Faradaic reactions that occur at the electrode surface, and are presented below [37,38,39]:

(3) $$Co_{3}O_{4}+OH^{-}+H_{2}O↔3CoOOH+e^{-}$$

(4) $$CoOOH+OH^{-}↔CoO_{2}+H_{2}O+e^{-}$$

The GCD curve of the Co_3_O_4_-NF electrode is shown in Figure 6b. The GCD curve exhibits a nearly symmetric nature with the same charging and discharging times and a low value of internal resistance [39]. Figure 6c displays *C_s_* observed at various current densities. The *C_s_* value observed at 4 A/g is approximately 1032 F/g. The cyclic stability of the Co_3_O_4_-NF electrode studied for the 5000th CV cycle is presented in Figure 6d, and the inset shows the stability study at 5000 CV cycles for the Co_3_O_4_-NF thin film. The observed b-value is 0.45 at the cathodic potential, which is close to 0.5, rather than 1, as shown in Figure A3c. This indicates that the charge storage contributions originate from the dominant diffusion-controlled processes. Figure A3d shows the capacitive and diffusion-controlled contributions of the Co_3_O_4_-NF electrode. As the scan rate increased, the capacitive contribution to the total charge increased. A major diffusion-controlled process for the Co_3_O_4_-NF electrode was observed at all of the scan rates, which is consistent with the battery-type nature of the electrode during the charge/discharge process.

### 3.5. Supercapacitive Performance of the Solid-State Co_3_O_4_-NF//α-Fe_2_O_3_-NFASC Device

**Figure 7:**
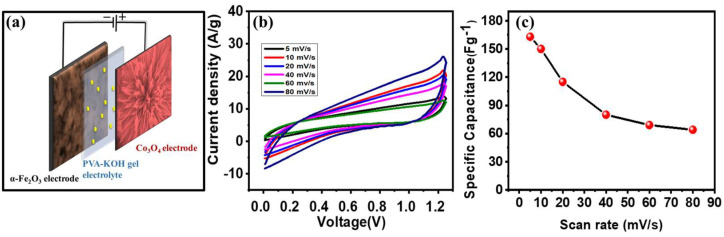
(**a**) Schematic of the supercapacitor device, (**b**) CV curves at different scan rates for the Co_3_O_4_-NF//α-Fe_2_O_3_-NF ASC device, (**c**) variation in specific capacitance at different scan rates for the Co_3_O_4_-NF//α-Fe_2_O_3_-NF ASC device.

**Figure 8:**
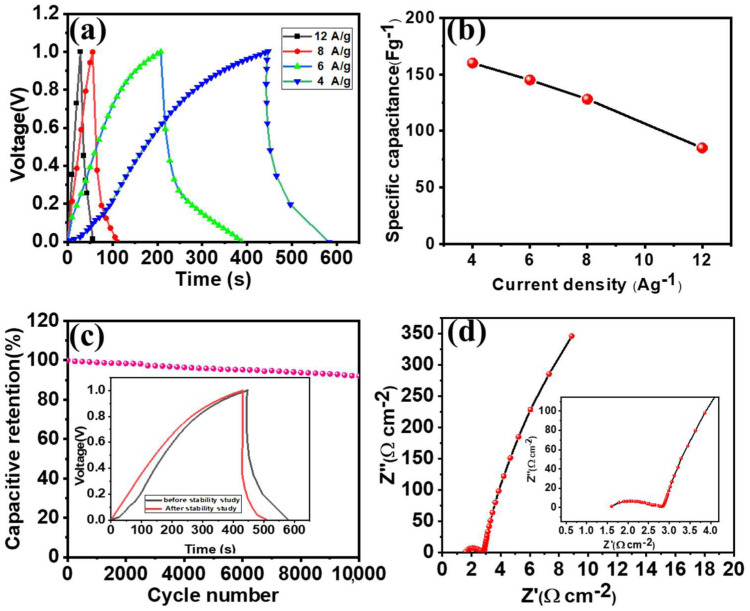
(**a**) GCD curves, (**b**) variation in specific capacitance at different current densities, (**c**) capacitive retention study, and inset show stability study, (**d**) Nyquist plot, and the inset shows the high-frequency region for the Co_3_O_4_-NF//α-Fe_2_O_3_-NF ASC device.

**Figure A4:**
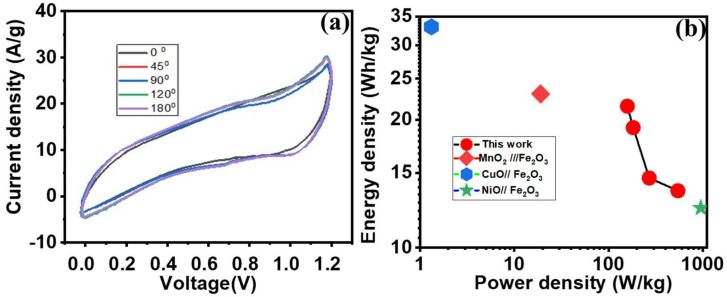
(**a**) CV curves at different angles, (**b**) the Ragone plot for the Co_3_O_4_-NF//Fe_2_O_3_-NF ASC device.

In this study, an ASC device was fabricated using Co_3_O_4_-NF and α-Fe_2_O_3_-NF as the positive and negative electrodes, respectively, with PVA-KOH as the solid-state electrolyte (Figure 7a). The charges across the Co_3_O_4_-NF and α-Fe_2_O_3_-NF electrodes were balanced using Equation (1). The mass loaded on both positive (Co_3_O_4_-NF) and negative electrodes (α-Fe_2_O_3_-NF) are 1.85 and 2.15 mg/cm^2^, respectively. Figure 7b shows the CV profile of the Co_3_O_4_-NF//α-Fe_2_O_3_-NF ASC device at various scan rates in the potential window of 0–1.0 V. The specific capacitance calculated for the Co_3_O_4_-NF//α-Fe_2_O_3_-NF device was approximately 164 F/g, as shown in Figure 7c. In the case of electrochemical double-layer capacitance, the ideal shape of the CV is rectangular, and the mirror image and current density are independent of the operating potential of the energy storage device during the discharge process [40]. The CV curves for the ASC device at different angles, such as 0°, 45°, 90°, 120°, and 180°, are shown in Figure A4a (Appendix A). The shape of the CV curve is not an ideal rectangular redox peak, and the shapes of the CV curves do not change [41,42,43]. The GCD curves for the Co_3_O_4_-NF//α-Fe_2_O_3_-NF ASC device at current densities of 4–12 A/g are shown in Figure 8a. The GCD curves show nearly symmetric charge–discharge curves. In addition, the voltage plateaus observed in all of the GCD curves confirm the contribution of the capacitance originating from the pseudocapacitive nature of the electrode [42]. The *C_s_* values calculated from the GCD curves were plotted as a function of the current density (Figure 8b). The device delivered a high *C_s_* of 160 F/g at 4 A/g. Figure A4b shows the Ragone plot of the energy density vs. power density for the fabricated Co_3_O_4_-NF//α-Fe_2_O_3_-NF ASC device. The maximum energy density of the device was observed up to 21.5 Wh/kg at a power density of 158.2 W/kg and a current density of 4 g^−1^. Table A2 in Appendix A shows the electrochemical performance of the ASC device, based on Co_3_O_4_-NF//α-Fe_2_O_3_-NF. The stability of the Co_3_O_4_-NF//α-Fe_2_O_3_-NF ASC device was tested using the GCD technique for 10,000 cycles. The capacitance retention of the Co_3_O_4_-NF//α-Fe_2_O_3_-NF ASC device is approximately 92%, as shown in Figure 8c. In addition, no significant difference was observed in the GCD curves before and after the stability study (Figure 8c). Furthermore, the electrochemical properties of the Co_3_O_4_-NF//α-Fe_2_O_3_-NF ASC device were studied using EIS. Figure 8d shows the Nyquist plot for the Co_3_O_4_-NF//α-Fe_2_O_3_-NF ASC device in the frequency range of 5 kHz to 50 MHz (5 mV), and the inset shows the high-frequency region. The obtained values of series resistance (*R_s_*) and charge transfer resistance (*R_ct_*) for the Co_3_O_4_-NF//α-Fe_2_O_3_-NF ASC device are 0.15 and 2.5 Ω/cm^2^, respectively. Thus, the ACS device based on Co_3_O_4_-NF//α-Fe_2_O_3_-NF exhibited good electrochemical properties.

## 4. Conclusions

Nanosphere-like α-Fe_2_O_3_ was uniformly deposited on an NF substrate using a simple CBD technique. The porous nature of α-Fe_2_O_3_ contributed to its good specific capacitance with enhanced cyclic stability. The Co_3_O_4_-NF//α-Fe_2_O_3_-NF device, fabricated using Co_3_O_4_-NF and α-Fe_2_O_3_-NF as the positive and negative electrodes, respectively, shows the maximum specific capacitance (*C_s_*) of 164 F/g at a current density of 4 A/g. The maximum energy density of the device was observed up to 21.5 Wh/kg at a power density of 158.2 W/kg, with excellent rate capability. In addition, the Co_3_O_4_-NF//α-Fe_2_O_3_-NF ASC device showed a capacitance retention of 92% after 10,000 cycles of GCD. The outstanding performance of α-Fe_2_O_3_-NF makes it one of the favorable negative electrode materials for high-performance energy storage devices.

## Data Availability

The data presented in this study are available on request from the corresponding author. The data are not publicly available.

## Appendix A

### Appendix A.1. Materials Characterization

The formation of α-Fe_2_O_3_-NF and Co_3_O_4_-NF was confirmed by X-ray diffraction (XRD, Bruker AXS D8 advance model) analysis. The surface morphology of α-Fe_2_O_3_-NF and Co_3_O_4_-NF was examined by field emission scanning electron microscopy (FE-SEM) and transmission emission electron microscopy (TEM). X-ray photoelectron spectroscopy (XPS) analysis of the powdered sample (~1–5 mg) of α-Fe_2_O_3_-NF was performed to determine the chemical composition and oxidation states (X-ray source: monochromatic AI Kα, ultimate energy resolution and It = 0.50 eV FWHM, Ag3d intensity curve).

### Appendix A.2. Electrochemical Characterization

The electrochemical performances of the α-Fe_2_O_3_-NF and Co_3_O_4_-NF electrodes were tested using a three-electrode system on a ZIVE SP2 battery cycler. In the case of the three-electrode system, α-Fe_2_O_3_-NF and Co_3_O_4_-NF electrodes (1 cm^2^) were used as the working electrodes, platinum wire as the counter electrode, and Ag/AgCl as the reference electrode. The electrochemical properties were tested in a 2 M KOH electrolyte. The specific capacity (*Q_s_*) and specific capacitance (*C_s_*) were calculated from the discharge curve using the following formulas [43]:

(A1) $$Q_{s}=\frac{\int i\left(A\right)\times dt\left(s\right)}{m\times 3600}$$

(A2) $$C_{s}=\frac{Q_{s}\times 3600}{\Delta V}$$

where *i*, Δ*t*, *m*, and Δ*V* are the discharge current density (*A*), discharge time (*s*), mass of the active material (*g*), and potential window (*V*), respectively.

### Appendix A.3. Asymmetric Supercapacitor Device Based on α-Fe2O3-NF//Co3O4-NF

To fabricate a flexible asymmetric supercapacitor device, α-Fe_2_O_3_-NF and Co_3_O_4_-NF electrodes were used as the negative and positive electrodes, respectively. PVA-KOH was used as the gel polymer electrolyte and separator. The PVA-KOH electrolyte was prepared using a previously reported method [44]. The α-Fe_2_O_3_-NF and Co_3_O_4_-NF electrodes were painted with the PVA-KOH electrolyte and allowed to solidify at room temperature. This process was repeated 2 to 3 times to ensure that a sufficient amount of electrolyte was coated on the electrode surface. After the solidification process, the two electrodes were sandwiched, and the device was packed and tested. The Cs, energy density, and power density values were calculated using a previously reported formula [42]. The charges between the cathode and anode can be balanced for excellent electrochemical results for the ASC device using the theory of mass balance as per the following equation:

(A3) $$\frac{m_{+}}{m_{-}}=\frac{C_{-}\times \Delta V_{-}}{C_{+}\times \Delta V_{+}}$$

where *m*_(+ or −)_, Δ*V*_(+ or −)_, and *C*_(+ or −)_ are the mass of the active material (*g*), potential window (Δ*V*), and *C_s_* (F/g) of the positive and negative electrodes, respectively. The mass ratio calculated between the positive and the negative electrodes is 1:1.56. The specific capacitance (*C_sc_*, F/g), specific energy (*E*, Wh/kg), and specific power (*P*, W/kg) of the ASC cell were calculated using the following equations:

(A4) $$C_{sc}=\frac{I\times \Delta t}{m\times \Delta V}$$

(A5) $$E=\frac{0.5\times C_{sc}\times \Delta V^{2}}{3.6}$$

(A6) $$P=\frac{E\times 3600}{\Delta t}$$

**Table A1:** The electrochemical performance of the ferric-oxide-based negative electrode.

Material	Electrolyte	Capacitance	Stability	Ref.
Fe_2_O_3_/CF	5 M LiCl	180.4 mF/cm^2^	-	[44]
Fe_2_O_3_-C	3 M KOH	247.5 mAh/g (2 mV/s)	64% (5000)	[42]
Fe_2_O_3_ NF	5 M LiCl	145.9 mF/cm^2^ 10 mA/cm^2^	87.2% (5000)	[45]
Fe_2_O_3_@C	6 M KOH	304.9 at 1 A/g	90.7% (2000)	[46]
CF-Fe_2_O_3_	2 M KOH	1.56 F/cm^2^ at 10 mA/cm^2^	102% (5000)	[47]
Fe_2_O_3_/CF	0.5 M LiClO_4_	261 F/g at 1 A/g	82.7% (10,000)	[48]
Fe_2_O_3_/CF	2 M KOH	908 F/g at 10 A/g	90% (5000)	[36]
Fe_2_O_3_-NF	2 M KOH	2125 F/g at 4 A/g	95.2% (5000)	Present work

**Table A2:** The electrochemical performance of the ferric-oxide-based ASC devices.

ASC Devices	Electrolyte	Specific Capacitance	Energy Density	Power Density	Ref.
CuO//Fe_2_O_3_	CMC-Na_2_SO_4_	79 F/g	23 Wh/kg	19 kW/kg	[49]
MnO_2_@CuO//Fe_2_O_3_@C	PVA-LiCl	2.46 F/cm^3^ (0.13 A/cm^2^)	0.85 mWh/cm^3^	0.1 W/cm^3^	[50]
CF-Co_3_O_4_//CF-Fe_2_O_3_	PVA-KOH	17.5 F/cm^3^ (6 mA/cm^2^)	6.75 mWh/cm^3^	104 mW/cm^3^	[51]
MnO_2_///Fe_2_O_3_	PVA-LiClO_4_	74 F/g (5 mV/s)	33.1 Wh/kg	1.32 kW/kg	[44]
MnO_2_//Fe_2_O_3_	PVA-LiCl	1.21 F/cm^3^ (0.5 mA/cm^2^)	0.41 mWh/cm^3^	-	[51]
MnO_2_/CF//Fe_2_O_3_/CF	PVA-LiCl	1.5 F/cm^3^ (0.5 mA/cm^2^)	0.55 mWh/cm^3^	-	[44]
NiO//Fe_2_O_3_	PVA KOH	57.2 F/g	12.4 Wh/kg	951 W/kg	[32]
Co_3_O_4_-NF//Fe_2_O_3_-NF	PVA KOH	155 F/g	21.5 Wh/kg	158 kW/kg	Present work
